# Prevalence of High-Risk Pregnancy Among Pregnant Women Attending Antenatal Care Camps in Primary Health Centres in Kinaye and Vantamuri and Their Sub-Centres

**DOI:** 10.7759/cureus.27355

**Published:** 2022-07-27

**Authors:** Austin R Gomindes, Resya Bhakthavalsalan, Utkarsh Sharma, Shannon L Johnston, Ashiq Naushad

**Affiliations:** 1 School of Medical and Dental Science, University of Birmingham, BIrmingham, GBR; 2 Major Trauma Services, Queen Elizabeth Hospital Birmingham, Birmingham, GBR; 3 Higher Education Academy, Advanced Higher Education - UK Professional Standards Framework, Yorkshire, GBR; 4 Major Trauma Services, Queen Elizabeth Hospital Birmingham, BIrmingham, GBR; 5 Emergency Medicine, Queen Elizabeth Hospital Birmingham, Birmingham, GBR; 6 Nursing, Birmingham City University, Birmingham, GBR; 7 Internal Medicine, Sri Ramaswamy Memorial (SRM) Institute for Medical Sciences, Chennai, IND

**Keywords:** antenatal care visits, antenatal screening, pregnancy risk factors, high-risk pregnancy, : antenatal care

## Abstract

Introduction

Identifying pregnancy-related complications and high-risk pregnancies early on and effectively managing care for these pregnant women through a holistic approach guided by the main objectives of antenatal care (ANC) and efficient, good-quality health care through ANC services can reduce the risk of pregnancy-related complications, being favourable for both mother and child. This study was intended to identify the percentage of pregnant women in high-risk groups attending ANC clinics.

Objective

This study’s aim was to understand the prevalence of high-risk pregnancies in women attending ANC camps in Kinaye and Vantamuri villages in Belagavi, India.

Methods

A community-based cross-sectional study was carried out in a primary healthcare (PHC) setting that included all pregnant women attending antenatal camps and residing in the same areas. With a total study sample size of 200, consisting of pregnant women attending ANC camps in Kinaye and Vantamuri PHC, the data were collected using a pre-tested, pre-designed questionnaire after obtaining written informed consent from the participating pregnant women.

Results

The data were analysed using a chi-square test to uncover the relationship between socio-demographics, obstetric history, medical variables, and high-risk pregnancy. The results demonstrated that 48.5% of women attending ANC clinics were in the high-risk pregnancy group.

Conclusion

Pregnancy outcomes are greatly affected by a woman’s socio-demographic, obstetric, and medical variables. The WHO recommends a minimum of four ANC visits to safely identify and mitigate the risks and complications of high-risk pregnancy to ensure positive outcomes for both mothers and children.

## Introduction

Pregnancy is the time during which one or more offspring develops inside a woman. Antenatal care (ANC) is an umbrella term used to describe the medical procedures and care carried out during pregnancy [1]. In India, 21% of pregnant women used full pregnancy care on average, ranging from 2.3% to 65.9% across all states. Overall, 51.6% had four or more antenatal visits, 30.8% used active ingredients for at least 100 days, and 91.1% received one or more doses of tetanus toxoid immunisation [2]. However, the overall use of prenatal care was uneven; this study was designed to analyse these factors in two primary healthcare (PHC) centres. Receiving ANC for the duration of pregnancy no longer assures the benefit of interventions that might be effective in enhancing maternal health. Receiving ANC a minimum of four times, which is usually recommended by the WHO, will increase the chances of receiving effective and efficient maternal care for the duration of the antenatal visits [3].

Pregnancy is the time during which one or more offspring develops inside a woman. ANC is an umbrella term used to describe the medical procedures and care carried out during pregnancy [1]. In India, 21% of pregnant women used full pregnancy care on average, ranging from 2.3% to 65.9% across all states. Overall, 51.6% had four or more antenatal visits, 30.8% used active ingredients for at least 100 days, and 91.1% received one or more doses of tetanus toxoid immunisation [2]. However, the overall use of prenatal care was uneven; this study was designed to analyse these factors in two primary healthcare centres. Receiving ANC for the duration of pregnancy no longer assures the benefit of interventions that might be effective in enhancing maternal health. Receiving ANC a minimum of four times, which is usually recommended by the WHO, will increase the chances of receiving effective and efficient maternal care for the duration of the antenatal visits [3].

The main objectives of the ANC are as follows: (i) maintenance of the health of the mother during pregnancy; (ii) identification of high-risk cases and appropriate management; (iii) prevention of development of complications; (iv) decrease of maternal and infant mortality and morbidity; (v) removal of the stress and worries of the mother regarding the delivery process; (vi) education of the mother about child-care/nutrition/sanitation/hygiene; (vii) advice about family planning; (viii) care of children under five years old accompanying pregnant mothers.

A high-risk pregnancy is defined as one complicated by a factor or factors adversely affecting the pregnancy outcome (maternal, perinatal, or both) [4]. Although only 10-30% of the mothers seen in the antenatal period could be classified as high risk, they account for more than 70% of the perinatal mortality and morbidity among mothers studied [2]. Every year, nearly 500,000 women die globally because of pregnancy-related causes. For each death, nearly 118 women suffer from life-threatening events or severe acute morbidity [5]. Perinatal outcomes can be changed significantly through early detection and special intensive care for high-risk pregnancies. All pregnancies should therefore be screened for the presence of risk factors. Factors to be considered include age, parity, social class, history of chronic disease (diabetes mellitus, hypertension, heart disease, thyroid disease, etc.), history of previous pregnancy complications, and multiple previous pregnancies. Early age of conception and frequent pregnancies, compounded with close spacing, contribute to higher perinatal mortality and morbidity, which can cause adverse health consequences for both mothers and children [6,7]. Hence, adequate (a minimum of four visits, as recommended by the WHO) ANC should be done to identify high-risk pregnancies at an early stage and manage any pregnancy-related complications to ensure acceptable maternal and perinatal outcomes. The objectives of the present study were to identify the prevalence of high-risk pregnancy among antenatal women and to determine the association between socio-demographic factors and high-risk pregnancy [3,5].

Objectives of the study

This study’s aim is to determine the prevalence of high-risk pregnancies in women attending ANC camps in Kinaye and Vantamuri villages in Belagavi, India.

## Materials and methods

Type of study

This is a community-based cross-sectional study.

Study setting

This study was carried out in Kinaye PHC (Karle, Khadawadi, Macche 1 and 2, Peeranwadi) and Vantamuri (Kakati 1 and 2, Bhutramanahatti, Honaga), which are the rural field practice areas of the Department of Community Medicine of Jawaharlal Nehru Medical College, Belagavi, Karnataka, India. ANC camps are organised by the tertiary care center because these women are placed so remotely that they have to travel for days to be able to access any health care. Hence, we organise a health camp and an ANC camp to get the required healthcare closer to them.

Inclusion criteria

All pregnant women attending ANC camps residing in the same areas were enrolled in the study. This study had a sample size of 200 pregnant women attending ANC camps in Kinaye and Vantamuri PHC.

Data collection

Pregnant women were interviewed using a pre-tested, pre-designed questionnaire (Appendix Figure 1) after the participants gave written informed consent. Information regarding socio-demographic variables, details of ANC care with the help of ANC records, and other factors (e.g., weight, height, previous obstetric history, health problems in the current pregnancy, general physical examination, and systemic examination) were collected. Blood investigations like haemoglobin, blood group, and RH typing were conducted. High-risk pregnancies were identified (e.g., teen pregnancy, elderly pregnancy, low socio-economic status, short stature, multiple pregnancies, multiple gestations, recurrent abortion, diabetes mellitus, hypertension and its related conditions, heart disease, epilepsy, thyroid disease, bronchial asthma, hepatitis B, antepartum haemorrhage, infection, prolonged labour, obstructed labour, postpartum haemorrhage, previous low socio-economic status, previous low birth weight, rh incompatibility, previous birth defects, and severe anaemia).

## Results

Statistical analysis 

The prevalence of high-risk pregnancies was 48.5%. The study was carried out for a period of one month, from December 1, 2018 to December 31, 2018. The data were analysed using a chi-square test to find the relationship between socio-demographic, obstetric, and medical variables and high-risk pregnancy.

According to the data collected and illustrated in Table 1, the largest cohort of pregnant women was in the 22-25 year age group, and there were no pregnant women above the age of 35 years.

**Table 1:** Age-wise distribution of women

Age (years)	Vantamuri		Kinaye	
	Number	Percentage	Number	Percentage
<18	1	1	0	0
18–21	31	31	20	20
22–25	40	40	49	49
26–30	23	23	27	27
31–35	5	5	4	4
>35	0	0	0	0

According to the data collected and analysed in Table 2, the distribution based on religion showed that most individuals followed Hinduism, followed by Islam.

**Table 2:** Religion-wise distribution of pregnant women

Religion	Vantamuri		Kinaye	
	Number	Percentage	Number	Percentage
Hindu	87	87	85	85
Muslim	13	13	15	15
Other	0	0	0	0

According to the data collected and analysed in Table 3, most individuals belonged to class 3 of the modified BG Prasad Classification of socio-economic status, and a minor amount of <5% belonged to classes 1 and 5.

**Table 3:** Socio-economic distribution of pregnant women

Socio-economic status (modified BG Prasad)	Vantamuri		Kinaye	
	Number	Percentage	Number	Percentage
1	3	3	2	2
2	39	39	35	35
3	39	39	40	40
4	14	14	17	17
5	5	5	6	6

According to the data collected and analysed in Table 4, most families were nuclear in nature.

**Table 4:** Family type distribution of pregnant women

Type of family	Vantamuri		Kinaye	
	Number	Percentage	Number	Percentage
Nuclear	28	28	91	91
Joint	72	72	9	9
Others	0	0	0	0

According to the data collected and analysed in Table 5, a significant number of pregnant women were married to men in their own near or distant families.

**Table 5:** Consanguinity distribution of pregnant women

Consanguineous	Vantamuri		Kinaye	
	Number	Percentage	Number	Percentage
Consanguineous	17	17	22	22
Non-consanguineous	83	83	78	78

According to the data collected and analysed in Table 6, most pregnant women were in the height group of >150 cm, and a very meagre percentage of pregnant women were <140 cm in height.

**Table 6:** Height distribution of pregnant women

Height (cm)	Vantamuri		Kinaye	
	Number	Percentage	Number	Percentage
<141	3	3	5	5
141–150	23	23	56	56
>150	74	74	39	39

According to the data collected and analysed in Table 7, most pregnant women were >45 kg in weight.

**Table 7:** Weight distribution of pregnant women

Weight (kg)	Vantamuri		Kinaye	
	Number	Percentage	Number	Percentage
<41	6	6	9	9
41–45	6	6	23	23
>45	88	88	68	68

According to the data collected and analysed in Table 8, most pregnant women attending ANC camps were in the second trimester of their pregnancy.

**Table 8:** Trimester distribution of pregnant women

Trimester	Vantamuri		Kinaye	
	Number	Percentage	Number	Percentage
First	12	12	8	8
Second	47	47	53	53
Third	41	41	39	39

According to the data collected and analysed in Table 9, most pregnant women attend ANC camps/clinics regularly.

**Table 9:** Distribution based on the number of antenatal care visits of pregnant women

ANC visits	Vantamuri		Kinaye	
	Number	Percentage	Number	Percentage
Adequate	86	86	53	53
Inadequate	14	14	47	47

According to the data collected and analysed in Table 10, most pregnant women attend ANC camps/clinics regularly.

**Table 10:** Distribution based on ante-natal USG scan of pregnant women done

USG scan	Vantamuri		Kinaye	
	Number	Percentage	Number	Percentage
Yes	95	95	88	88
No	5	5	12	12

According to the data collected and analysed in Table 11, all pregnant women were informed and prescribed and were compliant with the consumption of folic acid supplements.

**Table 11:** Distribution based on the number of pregnant women who had taken folic acid supplements

Folic acid	Vantamuri		Kinaye	
	Number	Percentage	Number	Percentage
Yes	98	98	95	95
No	2	2	5	5

According to the data collected and analysed in Table 12, all pregnant women were informed and prescribed and were compliant with the consumption of iron supplements.

**Table 12:** Distribution based on the number of pregnant women who had taken iron supplements

Iron	Vantamuri		Kinaye	
	Number	Percentage	Number	Percentage
Yes	96	96	91	91
No	4	4	9	9

According to the data collected and analysed in Table 13, all pregnant women were informed and prescribed and were compliant with the consumption of calcium supplements.

**Table 13:** Distribution based on the number of pregnant women who had taken calcium supplements

Calcium	Vantamuri		Kinaye	
	Number	Percentage	Number	Percentage
Yes	96	96	88	88
No	4	4	12	12

According to the data collected and analysed in Table 14, all pregnant women were informed and vaccinated with a minimum of one dose of TT immunisation.

**Table 14:** Distribution based on the number of pregnant women who had taken TT injections

TT injection	Vantamuri		Kinaye	
	Number	Percentage	Number	Percentage
Yes	98	98	90	90
No	2	2	10	10

According to the data collected and analysed in Table 15, most pregnant women did not have a history of complications in previous pregnancies or were primigravida.

**Table 15:** Distribution based on the number of pregnant women who had had previous pregnancy compilations

Previous pregnancy complication	Vantamuri		Kinaye	
	Number	Percentage	Number	Percentage
Yes	9	9	26	26
No	64	64	39	39
Not applicable	27	27	35	35

According to the data collected and analysed in Table 16, there were no pregnant women with severe anaemia, whereas most of the pregnant women attending ANC camps had an HB value of >11 g/dl.

**Table 16:** Distribution based on the grade of anaemia in pregnant women

Grade of anaemia (WHO)	Vantamuri		Kinaye	
	Number	Percentage	Number	Percentage
Normal (>11 g/dl)	58	58	43	43
Mild (10.1–11 g/dl)	22	22	33	33
Moderate (7.1–10 g/dl)	20	20	24	24
Severe (<7 g/dl)	0	0	0	0

According to the data collected and analysed in Table 17, only <10% of pregnant women attending ANC camps had any known previous or recently detected medical complications.

**Table 17:** Distribution based on the number of pregnant women who were at high risk due to medical complications

Medical complication	Vantamuri		Kinaye	
	Number	Percentage	Number	Percentage
Yes	8	8	11	11
No	92	92	89	89

According to the data collected and analysed in Table 18, a significant number of pregnant women attending ANC camps had previous or recently detected obstetric complications.

**Table 18:** Distribution based on the number of pregnant women who were at high risk due to obstetric complications

Obstetrical complication	Vantamuri		Kinaye	
	Number	Percentage	Number	Percentage
Yes	25	25	63	63
No	75	75	37	37

According to the data collected and analysed in Table 19, Vantamuri PHC, along with its subcentres, showed that 30% of the women attending ANC camps had high-risk factors, and 67% of those attending ANC camps in Kinaye PHC and its subcenters had high-risk factors. For the mean, 48.5% of women attending ANC camps in Kinaye and Vantamuri and its subcentres had either medical or obstetric or both forms of high-risk factors.

**Table 19:** Type of high-risk distribution of pregnant women

High-risk pregnancy	Vantamuri		Kinaye	
	Number	Percentage	Number	Percentage
Yes	30	30	67	67
No	70	70	33	33

## Discussion

It was observed that 45.5% of the antenatal mothers were in the age group of 22-25 years, 86% of the mothers were Hindu, and 17.5% had complications in previous pregnancies. The study revealed an average of 48.5% of the mothers were at high risk.

The above results were supported by the study conducted in 2015 [8] on the prevalence of high-risk pregnancy factors among 150 pregnant women selected by a convenience sampling technique while visiting the antenatal OPD of a selected hospital in Punjab. A structured questionnaire was used to assess socio-demographic variables along with prevalence factors. It was found that 30% of the women were at high risk.

Another study was conducted to assess the prevalence of high-risk pregnancies among antenatal mothers. The study was conducted among 100 antenatal mothers chosen by a convenience sampling technique in the Obstetrics and Gynecological Outpatient Department, Chettinad Hospital and Research Institute, Kanchipuram district, India. The study revealed that 25% of the mothers were high-risk [9].

A further study [10] conducted community-based research to determine the prevalence and correlates of high-risk pregnancy in rural Haryana. The objectives of this study were to determine the prevalence and correlates of high-risk pregnancy in a rural block of Haryana. A cross-sectional study was carried out in all the 20 sub-centres under the Community Health Centre (Block Lakhanmajra), the rural field practise area of the Department of Community Medicine, PGIMS, Rohtak, from July 2011 to June 2012. Assuming the prevalence of high-risk pregnancy was 10%, 900 eligible subjects were taken into consideration as part of the sample. All registered pregnant women at the subcentre at that point in time were included. A pre-tested semi-structured interview schedule was used when interviewing the study subjects. Data were collected, compiled, and analysed using statistical software (SPSS version 20.0, IBM Corp., Armonk, NY). Overall, the prevalence of high-risk pregnancy was 131.4%.

Kumar and Gnanadeep were able to conclude that the prevalence of high-risk pregnancy in rural Dharwad was significant. Although only 10-30% of the mothers seen in the antenatal period could be classified as high risk, they accounted for 70-80% of the population’s perinatal mortality and morbidity. The aim of the study was to determine the prevalence of high-risk pregnancies and their association with socio-demographic factors in rural field practise areas associated with the Department of Community Medicine, DM College of Medical Sciences and Hospital, Dharwad. Pregnant women attending health centres in that area were the participants in the study. Data were collected from August 1, 2013 to October 31, 2013. A pre-designed, pre-tested proforma was used to collect information regarding socio-demographic characteristics and obstetric history. Height, weight, and BP were recorded; general physical and systemic examinations were done. Haemoglobin estimation was done by Sahli's method. The results showed that the prevalence of high-risk pregnancy was found to be 37% [9].

## Conclusions

Pregnancy outcomes are greatly affected by a woman’s socio-demographic, obstetric, and medical variables. The WHO recommends a minimum of four ANC visits to mitigate the risks of a high-risk pregnancy by providing access to interventions vital to their health and well-being and that of their infants; this should ideally be done by skilled health personnel, which refers to workers/attendants who are accredited health professionals-such as midwives, doctors, or nurses-who have been educated and trained to proficiency in the skills needed to manage normal (uncomplicated) pregnancies, childbirth, and the immediate postnatal period, as well as in the identification, management, and referral of complications in women and new-borns. Raising awareness has helped increase ANC attendance and use through the empowerment of pregnant women and the education of their husbands.

## Figures and Tables

**Figure 1:**
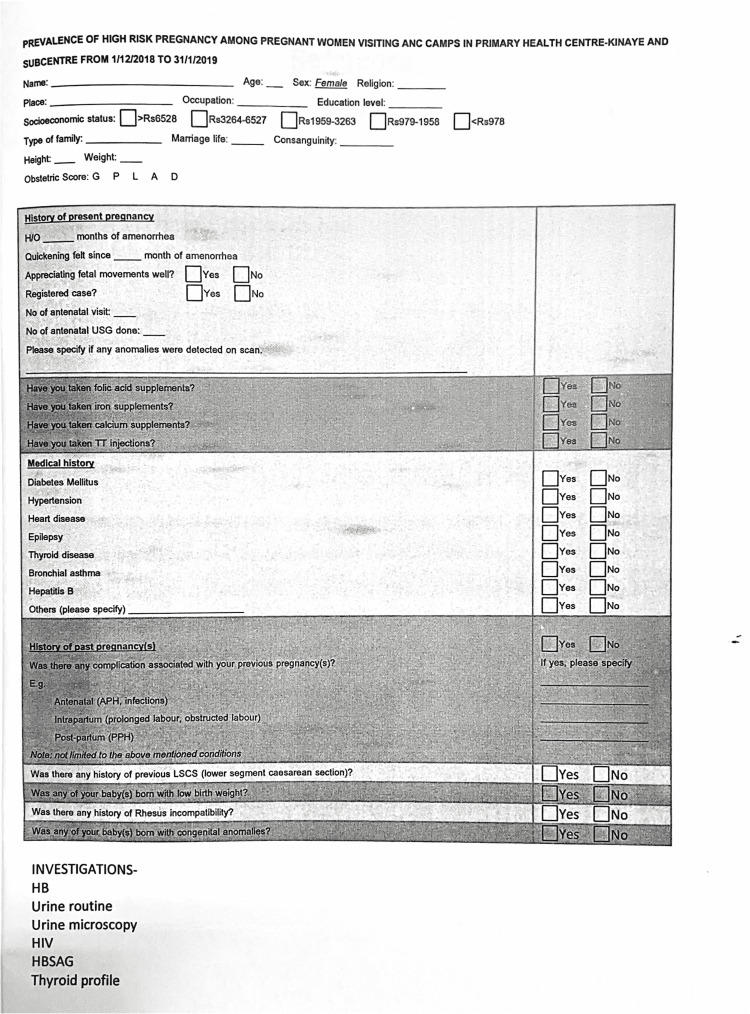
Pre-designed and pre-tested questionnaire
